# Short and long-term predictors of pain severity and interference in primary care patients with chronic musculoskeletal pain and depression

**DOI:** 10.1186/s12891-023-06357-2

**Published:** 2023-04-05

**Authors:** Concepció Rambla, Enric Aragonès, Meritxell Pallejà-Millán, Catarina Tomé-Pires, Germán López-Cortacans, Elisabet Sánchez-Rodríguez, Jordi Miró

**Affiliations:** 1 Institut Universitari d’Investigació en Atenció Primària (IDIAP) Jordi Gol, Barcelona, Spain; 2 Atenció Primària Camp de Tarragona, Institut Català de la Salut, Tarragona, Spain; 3 Centre d’Atenció Primària Constantí, Carrer dels Horts, 6, Constantí, 43120 Tarragona Spain; 4 Psychology Research Center (CIP), Autonomous University of Lisbon, Lisboa, Portugal; 5 Unit for the Study and Treatment of Pain – ALGOS, Research Center for Behavior Assessment (CRAMC), Department of Psychology, Universitat Rovira i Virgili, Catalonia, Spain

**Keywords:** Primary Health Care, Chronic pain, Major depression, Catastrophizing, Longitudinal studies

## Abstract

**Background:**

Chronic pain and depression are frequent comorbidities in primary care. Depression among other psychosocial factors play a role in the clinical course of chronic pain.

**Objective:**

To study the short and long-term predictive factors of severity and interference of chronic pain in primary care patients with chronic musculoskeletal pain and major depression.

**Methods:**

Longitudinal study of a cohort of 317 patients. The outcomes are severity and functional interference of pain (Brief Pain Inventory) measured at 3 and 12 months. We performed multivariate linear regression models to estimate the effects the explanatory baseline variables on the outcomes.

**Results:**

83% participants were women; average age was 60.3 years (SD = 10.2). In multivariate models, baseline pain severity predicted pain severity at 3 months (β = 0.53; 95% CI = 0.37–0.68) and at 12 months (β = 0.48; 95% CI = 0.29–0.67). Also, pain > 2 years of evolution predicted long term pain severity (β = 0.91; CI95%=0.11–1.71). Baseline pain interference predicted interference at 3 and 12 months (β = 0.27; 95%CI = 0.11–0.43 and β = 0.21; 95%CI = 0.03–0.40, respectively). Baseline pain severity predicted interference at 3 and 12 months (β = 0.26; 95%CI = 0.10–0.42 and β = 0.20; 95%CI = 0.02–0.39, respectively). Pain > 2 years predicted greater severity and greater interference at 12 months (β = 0.91; CI95%=0.11–1.71, and β = 1.23; CI95%=0.41–2.04). Depression severity predicted more interference at 12 months (β = 0.58; CI95%=0.04–1.11). Occupational status as active worker predicted less interference throughout the follow-up (β=-0.74; CI95%=-1.36 to -0.13 and β=-0.96; CI95%=-1.71 to -0.21, at 3 and 12 months). Currently working also predicts less pain severity at 12 months (β=-0.77; CI95%=1.52 − 0.02). With regard to the psychological variables, pain catastrophizing predicted pain severity and interference at three months (β = 0.03; 95% CI = 0.00-0.05 and β = 0.03; 95% CI = 0.00-0.05), but not at long term.

**Conclusion:**

In a sample of adults with chronic pain and depression, this primary care study has identified prognostic factors that independently predict the severity and functional interference of pain. If confirmed in new studies, these factors should be targeted for individualized interventions.

**Trial registration:**

ClinicalTrials.gov (NCT02605278), registered 16/11/2015.

**Supplementary Information:**

The online version contains supplementary material available at 10.1186/s12891-023-06357-2.

## Introduction

Musculoskeletal pain is one of the main causes of disability globally. It is estimated that chronic low back pain caused over 146 million disability-adjusted life years (DALYs) globally in 2013. Major depression was the second cause in this list, with 51 million DALYs. Other musculoskeletal conditions also in the top 10 causes of disability are chronic cervical pain and osteoarthritis [1]. The sustained upward trend of musculoskeletal pain from 1990 is expected to continue due to increasing life expectancy, elderly population, sedentary lifestyle and obesity [2], with a significant burden of suffering and disability in patients and important repercussions in the health systems and society [3, 4]. Chronic pain of musculoskeletal origin causes a third of all primary care consultations [5], and represents the main cause of disability [6].

In primary care, chronic pain and depression commonly present as comorbidities [7]. The relationship between chronic pain and depression is complex, it involves pathophysiological mechanisms and determines clinical expression. Moreover, it influences prognosis and response to treatment; that is to say, pain exacerbates the clinical course of depression and depression interferes in the management and response to pain treatment [8]. Research has shown that psycholosocial factors have a significant role in the adjustment to and coping with chronic pain. For example, there is mounting evidence showing that cognitive factors such as catastrophic thinking [9] and patients’ attitudes and beliefs towards pain are key to understand the severity and interference of chronic pain [10]. Most of this research has been conducted in individuals with chronic back pain in hospitals or occupational settings [11, 12]. It remains to be seen if the findings are also valid for patients in diverse locations (e.g., primary care facilities) were the caseload of patients and their characteristics will be different. A recent meta-analysis of studies on prognostic factors for musculoskeletal pain reported on the clinical importance of generic factors, not specific to a single anatomical location [13]. In primary care settings, widespread pain, that is, pain in multiple body areas, is more common than single pain [14]. In a previous study from our group, we already analyzed the associations between psychological variables and pain, with the limitation that it was a cross-sectional study [15]. Here, we go one step further and evaluate the predictive power of these variables on pain prognosis with a longitudinal study. Thus, the objective of this prospective study is to build on the body of knowledge on the predictors of pain in primary care patients, with the distinctive contribution of analyzing a sample of patients with comorbidity of chronic pain and depression. Increase our understanding of the implications of this frequent comorbidity [7] and the factors associated with the prognosis of musculoskeletal pain could help to improve the management of musculoskeletal conditions in these patients, and the design of new and better treatments [16] that address potentially modifiable factors [17].

## Methods

### Objective

To analyse which factors independently predict the severity and interference of short- and long-term chronic pain (3 and 12 months, respectively) in patients with chronic musculoskeletal pain and major depression in the primary care setting.

### Design

This study consists of a secondary analysis of the data from the DROP (DepRessiOn and Pain) study, a primary care clinical trial that evaluated a program for the integrated management of chronic pain and major depression in adults. The design and development of the DROP study has been detailed in previous publications [18, 19]. The protocol has been approved by the Institut d’Investigació en Atenció Primària (IDIAP) Jordi Gol Clinical Research Ethics Committee (P14/142) and is registered at ClinicalTrials.gov (NCT02605278).

In summary, it was a randomized clinical trial to investigate the effectiveness of a collaborative care model to improve the primary care clinical management of adult patients with chronic musculoskeletal pain and comorbid depression. The intervention included various components: (1) optimized management of depression using electronic clinical guidelines integrated into the primary care electronic medical records system; (2) a care manager that assisted the family doctors during patient monitoring and follow-up, and also accompanied the patients with follow-up and support by means of scheduled telephone calls; and (3) a group psychoeducational programme directed by the care manager that helped patients better understand their health and encouraged them to play an active role in the management of their pain, depression and associated difficulties. After 12 months, results showed that the programme improved depression compared to regular management, but no clinical benefits in pain outcomes were observed [19].

### Setting and participants

Participants were recruited from patient lists of 41 family physicians from primary care centres in the province of Tarragona (Catalonia, Spain). Inclusion criteria were as follows: age between 18 and 80 years; diagnosis of moderate or severe chronic musculoskeletal pain (Brief Pain Inventory pain severity scale ≥ 5 points) lasting over three months –according to a standard definition of chronic pain– [20], despite pain treatment; and meeting the diagnostic criteria of major depression at the time of inclusion (DSM-5 codes F32 or F33), ascertained by means of the major depression module of the SCID [21]. Exclusion criteria were any physical or mental limitation, or any comorbidity preventing participation in study evaluations (e.g., conditions such as severe deafness, cognitive impairment, intellectual disability, or serious physical illness), inability in Catalan and/or Spanish language, patients with a recorded diagnostic in his or her medical history of bipolar or somatization disorder, psychosis, fibromyalgia, alcohol or drug dependence, patients pregnant or breastfeeding, patients with an unresolved claim for occupational disability, and patients with a scheduled intervention for a prosthetic joint during the follow-up period.

### Measurements

The evaluations were conducted by an independent interviewer who administered in person a set of standardized questionnaires in the baseline assessment. Subsequently, the patients were monitored with telephone interviews during one year. In this study, we analyse the 3 and 12 month outcomes.

#### Outcomes: pain interference and severity

We used the 15-item version of the Brief Pain Inventory (BPI) to evaluate pain interference and severity [22, 23]. Pain severity is measured in several domains (worst pain, least pain, pain on average, and pain right now), using numerical scales from 0 (“no pain”) to 10 points (“worst pain”). The severity value is obtained from the average of the four domains. Interference is measured by scales from 0 (“no interference”) to 10 points (“total interference”) in seven areas of life: general activity, mood, walking, normal work, relationships, sleep, and enjoyment of life. The score on the interference scale is obtained by averaging the seven domains.

#### Baseline explanatory variables

##### Sociodemographic information

sex, age, marital status (single, married or living with a partner, divorced or separated, and widow or widower; dichotomized as living with a partner versus the other options), educational level (no formal education, primary school, lower secondary school, upper secondary school, and university; dichotomized as secondary or higher education versus the other options) and occupational situation (active, unemployed, permanent work disability, retired, and household chores; then dichotomized as current active working versus the other options).

##### Depression

Severity of depression was measured with the Hopkins Symptom Checklist, (HSCL-20) [24, 25], where the items investigate depressive symptoms scored on a Likert scale with five options, from 0 (“not at all”) to 4 (“extremely”). The overall score is the average of the 20 items. The medical history was checked for prior episodes of depression, and the duration of the current depressive episode was determined.

##### Pain characteristics

location (spine, limbs or both) and duration.

##### Catastrophic thinking

We used the Pain Catastrophizing Scale (PCS) [26, 27] to evaluate catastrophic thoughts related to pain. Catastrophizing is characterized by the perception of the painful stimulus as a threat, by the feeling of helplessness, and by being unable to avoid recurring thoughts related to pain. The PCS consists of 13 items scored on a Likert scale with 5 possible answers, from 0 (“this never happens to me”) to 4 (“it always happens to me”). The total score is the sum of the scores on all the items, and ranges between 0 and 52 points. The higher the scores, the greater the pain catastrophizing.

##### Attitudes towards pain

Beliefs or attitudes toward pain were measured with the Survey of Pain Attitudes (SOPA) [28, 29], which consists of 35 items with 5 answer options, from 0 (= totally false) to 4 (= totally true). The SOPA questionnaire investigates 7 dimensions: (1) control : to which extent the patient believes they can control their pain; (2) emotion: the patient believes that her or his emotions affect her or his experience of pain; (3) disability: the patient considers pain as a cause of disability; (4) harm: the patient beliefs that pain is a sign of physical injury and thus she or he should avoid exercise; (5) drugs: the patient beliefs that the pharmacological treatment is appropriate and effective for her or his pain; (6) solicitude: the extent to which the patient believes that others should be concerned for her or his pain; and (7) medical care: the patient believes that the physician is responsible for curing or improving her or his pain. Scores for each dimension, ranging from 0 to 4, were created by averaging item responses for each dimension, taking into account that some items have a reverse scoring.

##### Physical comorbidity

measured with the Duke Severity of Illness Checklist (DUSOI) [30, 31]. For each diagnosis, a score is assigned to the symptoms, complications, prognosis, and expected response to treatment. The overall severity of the patient is assessed on a scale of 0 to 100.

##### Comorbid anxiety

 evaluated using the anxiety section of the Primary Care Evaluation of Mental Disorders (PRIME-MD). This section includes three screening yes/no questions. If one of the answers is yes, the full diagnostic module must be applied. It consists of a structured interview that assesses panic disorder and generalized anxiety disorder diagnostic criteria [32, 33].

### Statistical analysis

We performed a descriptive analysis of the sample characteristics calculating means and standard deviations for continuous variables and percentages for categorical variables. To study the associations between the variables we calculated the baseline Spearman’s correlation coefficients.

The outcomes of the study were pain severity and pain interference, measured at 3 (short-term) and 12 months (long-term). We performed a bivariate linear regression to estimate the effects of each of the potentially explanatory baseline variables on the outcomes. The variables with p < 0.05 in the first analyses were then selected for the multivariate linear regression models. Only cases with complete data were considered in these analyses and no imputation methods of missing data were used.

Since we wanted to avoid any possible effect of the original clinical trial on the results, we included “study arm” as an adjustment variable in all multivariate models. A p value < 0.05 determined statistical significance. The statistical package R was used for all analyses.

## Results

The sample consisted of 317 patients, of which 272 (83%) were women and the average age was 60.3 years (SD = 10.2). Average pain severity was 6.52 (SD = 1.82) and the average functional impact was 6.35 (SD = 2.31), considering a score ≥ 7 as the cutting point between moderate and severe pain in both subscales. Most patients (84%) reported pain lasting over two years. Depression was chronic (> 2 years) in over half of the patients (58%), and the average severity was 1.68 points (SD = 0.74) (in the HSCL-20, the cutting point between moderate and severe depression is > 1.7) (Table 1).

**Table 1:** Baseline characteristics of study participants

Baseline features	
	n (%) ^1^
Age (mean and SD)	60.3 (10.2)
Gender: female	272 (82.9)
Marital status	
Single	15 (4.6%)
Married/ Partner	206 (63.6%)
Divorced/ Separated	52 (16.0%)
Widow/ Widower	51 (15.7%)
Level of education	
No formal education	42 (12.9%)
Primary school	172 (53.1%)
Lower secondary school	48 (14.8%)
Upper secondary school	48 (14.8%)
University	14 (4.3%)
Occupational status	
Active worker	64 (20.8%)
Unemployed	38 (12.3%)
Permanent work disability	26 (8.2%)
Retired	130 (41.0%)
Household chores	50 (16.2%)
Location of pain	
Spine	14 (4.3%)
Limb	18 (5.5%)
Both locations	298 (90.2%)
Severity of pain (BPI^2^ score; mean and SD)	6.52 (1.82)
Interference of pain (BPI^2^ score; mean and SD)	6.35 (2.31)
Duration of chronic pain ≥ 24 months	275 (83.8%)
Severity of depression (HSCL-20^3^ score; mean and SD)	1.68 (0.74)
Duration of depressive episode ≥ 24 months	190 (57.9%)
Recurrent depression	205 (62.9%)
Psychiatric comorbidity	
Panic disorder	95 (29.3%)
Generalised anxiety disorder	242 (74.0%)
Physical comorbidity (DUSOI^4^ score; mean and SD)	43.6 (11.8)
Survey of Pain Attitudes domains (SOPA; mean and SD)	
Control	2.15 (1.12)
Disability	1.41 (0.92)
Harm	1.94 (0.82)
Emotion	2.34 (1.10)
Medication	2.76 (0.98)
Solicitude	1.58 (1.43)
Medical cure	2.54 (1.03)
Pain Catastrophizing Scale (PCS; mean and SD)	1.68 (1.08)

^1^Unless stated otherwise; ^2^Brief Pain Inventory, providing scores for both pain severity and pain interference;^3^ Hopkins Symptom Checklist,20 items; ^4^Duke Severity of Illness Checklist

Of the 317 patients assessed at baseline, 305 were assessed at 3 months (4% dropout rate), and 274 at 12 months (14% dropout rate). As there were no differences between the patients who dropped out and those who remained in terms of age, gender, work condition, severity or pain interference, depression severity, or psychiatric or physical comorbidity, the patients who dropped out are considered to have done at random (Table A1, supplementary files).

When we analysed the correlation coefficients at baseline, we found linear relationships ranging from negligible to moderate. No strong correlations were found between the variables (Table A2, Supplementary files).

Throughout the follow-up, we observed great stability in the clinical expression of chronic pain, both in the severity of the pain and in the associated interference (Table 2).

**Table 2:** Pain severity and pain interference throughout the study period, according to Brief Pain Inventory (BPI) scores

	Baseline	3 months	12 months
	N = 328	N = 305	N = 274
Severity of pain (BPI^1^ score; mean and SD)	6.5 (1.8)	6.5 (2.4)	6.4 (2.6)
Interference of pain (BPI^1^ score; mean and SD)	6.3 (2.3)	5.6 (2.5)	5.5 (2.6)

^1^Brief Pain Inventory, providing scores for both pain severity and pain interference

### Bivariate analysis. Factors associated with pain severity and interference at 3 and 12 months

Table 3 shows the associations between baseline characteristics and pain severity at 3 and 12 months. Pain severity and interference at baseline, pain lasting over 2 years, and aspects related to psychiatric comorbidity were associated with greater pain severity at 3 and 12 months. Male gender and living with a partner were associated with lower severity of pain at three months. A higher educational level was associated with lower severity of pain at 3 and 12 months. Working predicted a better result at 12 months.

**Table 3:** Predictors of pain intensity at 3 and 12 months for patients with chronic musculoskeletal pain and co-morbid depression in the bivariate analysis

	3 months			12 months		
Baseline variables	β coefficient	95% CI	p	β coefficient	95% CI	p
Sociodemographic variables						
*Gender (male)*	-0.723	-1.416 to -0.031	0.041	-0.587	-1.446 to 0.272	0.179
*Age (years)*	0.013	-0.014 to 0.039	0.344	0.019	-0.012 to 0.050	0.222
*Lives with partner*	-0.645	-1.201 to -0.090	0.023	-0.045	-0.695 to 0.605	0.891
*Currently working*	-0.327	-1.003 to 0.350	0.342	-0.971	-1.744 to-0.197	0.014
*Secondary or higher education*	-0.708	-1.269 to -0.148	0.013	-0.661	-1.313 to -0.010	0.047
Brief Pain Inventory, intensity	0.688	0.561 to 0.816	< 0.001	0.617	0.462 to 0.772	< 0.001
Brief Pain Inventory, interference	0.400	0.294 to 0.505	< 0.001	0.336	0.208 to 0.464	< 0.001
Length of chronic pain ≥ 24 months	0.770	0.050 to 1.490	0.036	1.086	0.234 to 1.938	0.013
Psychiatric comorbidity						
*Length of depressive episode ≥ 24 months*	0.349	-0.189 to 0.886	0.203	0.188	-0.437 to 0.814	0.553
*Severity of depression: HSCL-20 score*^1^	0.772	0.412 to 1.133	< 0.001	0.669	0.234 to 1.103	0.003
*Anxiety disorders*	1.181	0.554 to 1.808	< 0.001	0.936	0.209 to 1.662	0.012
Physical comorbidity: DUSOI score^2^	0.008	-0.014 to 0.030	0.483	0.008	-0.018 to 0.034	0.527
Survey of Pain Attitudes (SOPA) domains						
*Control*	-0.332	-0.567 to -0.097	0.006	-0.054	-0.344 to 0.235	0.713
*Harm*	0.384	0.060 to 0.707	0.020	0.402	0.018 to 0.785	0.040
*Emotion*	0.478	0.240 to 0.715	< 0.001	0.470	0.196 to 0.744	0.001
*Disability*	0.784	0.505 to 1.063	< 0.001	0.562	0.222 to 0.901	0.001
*Medication*	0.123	-0.152 to 0.397	0.381	0.441	0.130 to 0.752	0.006
*Medical cure*	-0.003	-0.264 to 0.258	0.982	-0.136	-0.435 to 0.163	0.372
*Solicitude*	0.183	-0.003 to 0.369	0.054	0.324	0.109 to 0.538	0.003
Pain Catastrophizing Scale (PCS) global score	0.058	0.040 to 0.076	< 0.001	0.045	0.022 to 0.067	< 0.001
Study arm (intervention)	-0.076	-0.609 to 0.456	0.778	-0.433	-1.051 to 0.186	0.170

^1^ Hopkins Symptom Checklist, 20 items; ^2^ Duke Severity of Illness Checklist

Table 4 shows that both severity and interference at baseline are associated with pain interference at 3 and 12 months. The baseline severity of depression and anxiety disorders as comorbidities are associated with greater interference at 3 and 12 months. Health-related quality of life at baseline was associated with less interference at 3 and 12 months. Working was associated with less interference throughout.

**Table 4:** Predictors of pain interference at 3 and 12 months for patients with chronic musculoskeletal pain and co-morbid depression in the bivariate analysis

	3 months			12 months		
Baseline variables	β coefficient	95% CI	p	β coefficient	95% CI	p
Sociodemographic variables						
*Gender (male)*	-0.115	-0.855 to 0.625	0.760	-0.371	-1.233 to 0.492	0.398
*Age*	-0.016	-0.044 to 0.011	0.247	-0.008	-0.038 to 0.023	0.623
*Lives with partner*	-0.507	-1.097 to 0.084	0.092	-0.212	-0.862 to 0.439	0.522
*Currently working*	-0.711	-1.419 to -0.003	0.049	-1.015	-1.797 to -0.233	0.011
*Secondary or higher education*	-0.382	-0.980 to 0.216	0.210	-0.010	-0.667 to 0.647	0.977
Brief Pain Inventory, intensity	0.589	0.445 to 0.733	< 0.001	0.448	0.285 to 0.612	< 0.001
Brief Pain Inventory, interference	0.540	0.434 to 0.645	< 0.001	0.406	0.281 to 0.532	< 0.001
Length of chronic pain ≥ 24 months	0.645	-0.121 to 1.410	0.099	1.415	0.568 to 2.262	0.001
Psychiatric comorbidity						
*Length of depressive episode ≥ 24 months*	0.422	-0.148 to 0.992	0.146	0.347	-0.279 to 0.973	0.276
*Severity of depression: HSCL-20 score 1*	1.226	0.858 to 1.594	< 0.001	1.144	0.722 to 1.565	< 0.001
*Anxiety disorders*	1.217	0.551 to 1.882	< 0.001	1.177	0.453 to 1.900	0.002
Physical comorbidity: DUSOI score^2^	0.016	-0.008 to 0.039	0.195	0.003	-0.023 to 0.029	0.842
Survey of Pain Attitudes (SOPA) domains						
*Control*	-0.642	-0.885 to -0.400	< 0.001	-0.311	-0.599 to -0.023	0.034
*Harm*	0.634	0.295 to 0.972	< 0.001	0.381	-0.004 to 0.766	0.052
*Emotion*	0.679	0.432 to 0.925	< 0.001	0.361	0.084 to 0.639	0.011
*Disability*	1.171	0.890 to 1.452	< 0.001	0.891	0.560 to 1.221	< 0.001
*Medication*	0.178	-0.113 to 0.469	0.230	0.368	0.055 to 0.681	0.021
*Medical cure*	-0.038	-0.315 to 0.238	0.786	-0.248	-0.547 to 0.051	0.103
*Solicitude*	0.333	0.138 to 0.529	0.001	0.270	0.054 to 0.486	0.014
Pain Catastrophizing Scale (PCS) global score	0.079	0.061 to 0.097	< 0.001	0.049	0.027 to 0.071	< 0.001
Study arm (intervention)	-0.383	-0.946 to 0.180	0.182	-0.562	-1.181 to 0.057	0.075

^1^ Hopkins Symptom Checklist, 20 items; ^2^ Duke Severity of Illness Checklist

We analysed the associations of various psychological variables with outcomes (Tables 2 and 3). Catastrophizing was associated with both variables at 3 and 12 months. Some dimensions of SOPA show significant associations at 3 and 12 months.

As expected, since the original clinical trial did not show improvement on the evolution of pain, the arm of the clinical trial where the patient was allocated was not associated with any pain outcome during follow-up.

In linear regression models, baseline pain severity predicted greater pain severity at 3 and 12 months. Pain lasting over two years predicted greater long term severity. Actively working predicted less pain severity at 12 months (Table 5).

**Table 5:** Multivariate analysis to identify independent predictors of pain severity at 3 and 12 months for patients with chronic musculoskeletal pain and comorbid depression

	3 months^2^			12 months^3^		
Baseline variables	β coefficient	95% CI	p	β coefficient	95% CI	p
Sociodemographic variables						
*Gender (male)*	-0.355	-0.962 to 0.253	0.251	-	-	-
*Lives with partner*	-0.436	-0.915 to 0.043	0.074	-	-	-
*Currently working*	-	-	-	-0.770	-1.520 to -0.019	0.044
*Secondary or higher education*	-0.237	-0.730 to 0.256	0.346	-0.151	-0.776 to 0.475	0.636
Brief Pain Inventory, severity	0.525	0.367 to 0.682	< 0.001	0.483	0.295 to 0.670	< 0.001
Brief Pain Inventory, interference	0.111	-0.038 to 0.260	0.144	0.158	-0.028 to 0.344	0.095
Duration of chronic pain ≥ 24 months	0.504	-0.129 to 1.136	0.118	0.907	0.108 to 1.707	0.026
Psychiatric comorbidity						
*Severity of depression: HSCL-20 score*^1^	-0.054	-0.471 to 0.362	0.798	0.029	-0.495 to 0.554	0.912
*Anxiety disorders*	0.545	-0.060 to 1.150	0.077	0.520	-0.196 to 1.236	0.154
Survey of Pain Attitudes (SOPA) domains						
*Control*	0.249	-0.029 to 0.527	0.079	-	-	-
*Harm*	-0.183	-0.489 to 0.124	0.242	-0.071	-0.450 to 0.308	0.714
*Emotion*	-0.071	-0.315 to 0.171	0.564	0.039	-0.268 to 0.345	0.804
*Disability*	0.226	-0.134 to 0.587	0.218	-0.141	-0.561 to 0.278	0.508
*Medication*	-	-	-	0.239	-0.060 to 0.539	0.116
*Solicitude*	-	-	-	0.121	-0.097 to 0.338	0.275
Pain Catastrophizing Scale (PCS) global score	0.027	0.002 to 0.052	0.033	-0.007	-0.036 to 0.023	0.653
Study arm (intervention)	-0.025	-0.483 to 0.433	0.915	-0.350	-0.913 to 0.213	0.222

^1^ Hopkins Symptom Checklist, 20 items; 3 months model: F (14,285) = 10.851, Adj. R2 0.316; 12 months model: F (14,251) 6.470, Adj.R2 0.224

Table 6 shows the results with interference as outcome. Both baseline pain severity and interference predicted interference at 3 and 12 months. Pain lasting over two years and baseline severity of depression predicted a greater interference at 12 months. Actively working predicted less interference throughout the follow-up.

**Table 6:** Multivariate analysis to identify independent predictors of pain interference at 3 and 12 months for patients with chronic musculoskeletal pain and comorbid depression

	3 months^3^			12 months^4^		
Baseline variables	β coefficient	95% CI	p	β coefficient	95% CI	p
Sociodemographic variables						
*Currently working*	-0.741	-1.356 to -0.126	0.018	-0.956	-1.706 to -0.206	0.013
Brief Pain Inventory, severity	0.260	0.097 to 0.424	0.002	0.202	0.017 to 0.388	0.033
Brief Pain Inventory, interference	0.266	0.107 to 0.425	0.001	0.214	0.027 to 0.401	0.025
Duration of chronic pain ≥ 24 months	-	-	-	1.226	0.414 to 2.038	0.003
Psychiatric comorbidity						
*Severity of depression: HSCL-20 score*^1^	0.147	-0.294 to 0.588	0.512	0.577	0.044 to 1.111	0.034
*Anxiety disorders*	0.113	-0.521 to 0.746	0.727	0.432	-0.292 to 1.156	0.241
Survey of Pain Attitudes (SOPA) domains						
*Control*	0.142	-0.148 to 0.431	0.335	0.252	-0.087 to 0.591	0.145
*Harm*	-0.063	-0.386 to 0.260	0.700	-	-	-
*Emotion*	0.007	-0.257 to 0.271	0.961	-0.161	-0.473 to 0.150	0.309
*Disability*	0.402	0.020 to 0.784	0.039	0.285	-0.147 to 0.718	0.195
*Medication*	-	-	-	0.150	-0.149 to 0.450	0.324
*Solicitude*	-0.002	-0.191 to 0.186	0.981	0.027	-0.193 to 0.248	0.806
Pain Catastrophizing Scale (PCS) global score	0.027	0.000 to 0.054	0.047	0.004	-0.028 to 0.035	0.825
Study arm (intervention)	-0.294	-0.777 to 0.190	0.233	-0.517	-1.087 to 0.053	0.075

^1^ Hopkins Symptom Checklist, 20 items; 2Duke Severity of Illness Checklist; 3 months model: F (12,282) = 12.329, Adj.R2 = 0.316; 12 months model: F (13,252) 6.831, Adj. R2 0.222

Regarding the role of psychological variables as prognostic factors, we identified catastrophizing as a predictor of pain severity and interference at three months, but not long term (Tables 5 and 6). The SOPA domain ‘disability’ is associated with greater interference at 3 months. Our models did not identify any other attitudes and beliefs about pain measured by SOPA as predictive factors.

## Discussion

The objective of this primary care study is to analyse independent factors able to predict the severity and interference of short- and long-term chronic pain in patients with chronic musculoskeletal pain and major depression. We have identified various modifiable and non-modifiable factors associated with clinical pain outcomes. We have observed that the baseline severity and interference of pain is predictive of the short and long-term evolution of pain. Our results in patients with chronic pain and depression agree with the results of a recent meta-analysis [13] on the evolution of musculoskeletal pain in the primary care setting. This work also identified the severity and interference of baseline pain as prognostic factors in the evolution of pain. This meta-analysis also reports that generalized pain or pain in multiple locations might be a negative predictive factor. Although we did not identify this factor in our analyses, we note that in our study 90% of patients experienced pain at multiple sites.

In our study, working at baseline was identified as an independent predictor of a lesser interference of short- and long-term pain. This result must be interpreted cautiously because it is possible that patients with higher pain interference, and with a worse prognosis, had stopped working for this reason before the baseline assessment. Moreover, the interrelationship between occupational status and disability associated with chronic pain are complex and influenced by other clinical and socioeconomic factors [34, 35]. Studies have often reported a longitudinal association between not working with adverse pain outcomes [36, 37]. As suggested by other researchers [38], our results could align with the hypothesis that promoting job reinstatement in patients with chronic pain may benefit them.

Except for an isolated result in which baseline depression severity is independently associated with less long-term interference, our study does not confirm psychiatric comorbidities as predictive factors, although in previous studies they are emphasized as prognostic factors in the evolution of pain [39, 40]. The widespread presence of psychological pathology in our sample, where all patients have depression and the vast majority have anxiety disorders, may have prevented a more clear observation of a possible predictive effect of psychological variables in the course of pain.

Regarding cognitive variables, catastrophizing was associated with worse short-term outcomes, while personal attitudes or beliefs toward pain could not be considered independent predictors of pain severity and interference. Catastrophizing is a cognitive response to pain that amplifies feelings of pain, rumination, obsessive worrying and helplessness regarding our ability to cope with and manage our own pain [41]. While the literature regarding catastrophic thinking as a prognostic factor is controversial, it is usually associated with a worse evolution of pain [9, 42, 43]. Moreover, although catastrophizing correlates closely with depression, it has a specific and additive deleterious effect on the evolution of pain [43]. In contrast, a multicentre study to analyse the prognostic value of catastrophizing in a large sample of patients with low back pain in Spain concluded that catastrophizing at baseline did not predict the evolution of pain or associated disability [44]. However, since catastrophizing is potentially modifiable, our results strengthens the hypothesis that interventions to decrease catastrophizing could improve the prognosis of chronic pain [45], at least in the short term. However, this improvement has not always been confirmed in clinical trials [46].

In agreement with published research [47], our previous cross-sectional analysis [14] suggested that patients’ adaptive or maladaptive attitudes and beliefs toward pain could play a prognostic role in pain progression. The interest of this hypothesis lies in the possibility of modifying maladaptive beliefs and enhancing adaptive beliefs through psychological interventions [48]. However, our longitudinal results have not confirmed this hypothesis, beyond having identified the predictive value of the SOPA ‘disability’ subscale in short-term interference.

This study has potential limitations. Firstly, before interpreting the results, we should understand the characteristics of participants. A study by Meisingset et al. [49] concluded that the phenotype of patients with musculoskeletal pain characterized by intense, generalized pain, with significant long-term functional interference and psychological distress (characteristics of the majority of our patients) presented a worse evolution and lower response to interventions. In addition, the sample was recruited from a limited number of primary care centres from a specific area. All these factors can hinder the extrapolation of our results to patients with chronic musculoskeletal pain in other levels of care and from other geographical areas. Secondly, although current objectives are consistent and complementary with the original clinical trial, these conclusions originate from a secondary analysis of data collected for different purposes.

### Implications

Despite these limitations, our results provide valuable information on independent factors that predict pain severity and functional interference in a sample of primary care patients with chronic pain and depression. We have identified prognostic factors potentially modifiable with multifactorial interventions, such as catastrophic thinking and occupational status. It is also important to understand the non-modifiable prognostic factors, such as the baseline clinical status or previous evolution, to personalise management and avoid unrealistic expectations. Further studies should confirm the hypothesis that the modification of predictive factors translates into better pain outcomes.

## Electronic supplementary material

Below is the link to the electronic supplementary material.

Supplementary Material 1

Supplementary Material 2

## Data Availability

The datasets generated and analysed for this study are not publicity available due to privacy and ethical concerns but are available from the corresponding author on reasonable request.

## List of Abbreviations

BPI: Brief Pain Inventory
DSM-5: Diagnostic and Statistical Manual of Mental Disorders, Fifth Edition
DALY: Disability adjusted life years
DROP: Depression and Pain Project
DUSOI: Duke Severity of Illness Checklist
HSCL-20: Hopkins Symptom Checklist 20 items
IDIAP Jordi Gol: Primary Care Research Institute Jordi Gol (in Catalan *Institut d’Investigació en Atenció Primària Jordi Gol*)
PCS: Pain Catastrophizing Scale
PRIME-MD: Primary Care Evaluation of Mental Disorders
SOPA: Survey of Pain Attitudes
