# Supplementary material for: Short and long-term predictors of pain severity and interference in primary care patients with chronic musculoskeletal pain and depression
Source: BMC Musculoskelet Disord. 2023 Apr 5;24:270. doi: 10.1186/s12891-023-06357-2 (PMC10074832; doi:10.1186/s12891-023-06357-2)
Supplement: Supplementary file 1 — Supplementary Material 1 [file 12891_2023_6357_MOESM1_ESM.docx]

|  | 3 months | | | 12 months | | |
| --- | --- | --- | --- | --- | --- | --- |
|  | Remained | Dropouts | p value ^4^ | Remained | Dropouts | p value ^4^ |
|  | n= 305 | n= 12 |  | n= 274 | n= 43 |  |
| Age (mean and SD) | 60.3 (10.2) | 62.2 (9.05) | 0.506 | 60.4 (10.1) | 60.1 (10.4) | 0.860 |
| Gender: female (n and %) | 251 (82.3%) | 12 (100%) | 0.231 | 232 (84.7%) | 31 (72.1%) | 0.090 |
| Currently working (n and %) | 64 (20.3%) | 4 (33.3%) | 0.810 | 53 (19.9%) | 11 (26.8%) | 0.100 |
| Severity of pain (BPI^1^ score; mean and SD) | 6.51 (1.78) | 6.48 (1.76) | 0.942 | 6.51 (1.81) | 6.54 (1.58) | 0.915 |
| Interference of pain (BPI^1^ score; mean and SD) | 6.34 (2.31) | 6.59 (1.82) | 0.647 | 6.29 (2.31) | 6.73(2.16) | 0.220 |
| Severity of depression (HSCL-20^2^ score; mean and SD) | 1.67 (0.72) | 1.79 (0.83) | 0.632 | 1.67 (0.70) | 1.702 (0.85) | 0.828 |
| Psychiatric comorbidity |  |  |  |  |  |  |
| Panic disorder (n and %) | 89 (29.6%) | 3 (25.0%) | 1.000 | 79 (29.3%) | 13 (30.2%) | 1.000 |
| Generalised anxiety disorder (n and %) | 226 (74.3%) | 9 (75.0%) | 1.000 | 201 (73.63%) | 34 (79.07%) | 0.567 |
| Physical comorbidity (DUSOI^3^ score; mean and SD) | 43.4 (11.9) | 47.2 (8.9) | 0.175 | 43.9 (11.9) | 40.8 (11.3) | 0.098 |
| ^1^Brief Pain Inventory, providing scores for both pain severity and pain interference;^2^ Hopkins Symptom Checklist,20 items; ^3^Duke Severity of Illness Checklist; ^4^ Chi-squared test was used for categorical variables and the Student's T-test for continuous variables | | | | | | |

**Table A1. Analysis of the main characteristics of patients who dropped out at 3 and 12 months compared to those who remained**

|  | Survey of Pain Attitudes (SOPA) domains | | | | | | |  |  |  |  |  |
| --- | --- | --- | --- | --- | --- | --- | --- | --- | --- | --- | --- | --- |
|  | Control | Disability | Harm | Emotion | Medication | Solicitude | Medical cure | PCS^1^ | BPI^2^,  severity | BPI^2^,  interference | HSCL-20^3^ | DUSOI^4^ |
| Control | 1.000 | -0.545^***^ | -0.333^***^ | -0.259^***^ | -0.156^**^ | -0.245^***^ | 0.022 | -0.614^***^ | -0.272^***^ | -0.489^***^ | -0.396^***^ | -0.027 |
| Disability |  | 1.000 | 0.337^***^ | 0.396^***^ | 0.222^***^ | 0.335^***^ | -0.094 | 0.594^***^ | 0.324^***^ | 0.547^***^ | 0.466^***^ | 0.078 |
| Harm |  |  | 1.000 | 0.123^*^ | 0.064 | 0.081 | -0.087 | 0.292^***^ | 0.295^***^ | 0.334^***^ | 0.220^***^ | 0.074 |
| Emotion |  |  |  | 1.000 | 0.208^***^ | 0.375^***^ | 0.048 | 0.486^***^ | 0.280^***^ | 0.362^***^ | 0.369^***^ | 0.009 |
| Medication |  |  |  |  | 1.000 | 0.210^***^ | 0.107 | 0.280^***^ | 0.085 | 0.099 | 0.153^**^ | 0.177^**^ |
| Solicitude |  |  |  |  |  | 1.000 | 0.096 | 0.303^***^ | 0.095 | 0.222^***^ | 0.202^***^ | 0.081 |
| Medical cure |  |  |  |  |  |  | 1.000 | 0.040 | 0.020 | 0.074 | -0.063 | -0.180^**^ |
| PCS^1^ |  |  |  |  |  |  |  | 1.000 | 0.397^***^ | 0.580^***^ | 0.429^***^ | 0.028 |
| BPI^2^, severity |  |  |  |  |  |  |  |  | 1.000 | 0.508^***^ | 0.244^***^ | 0.052 |
| BPI^2^, interference |  |  |  |  |  |  |  |  |  | 1.000 | 0.585^***^ | 0.089 |
| HSCL-20^3^ |  |  |  |  |  |  |  |  |  |  | 1.000 | -0.003 |
| DUSOI^4^ |  |  |  |  |  |  |  |  |  |  |  | 1.000 |
| ^1^ PCS: Pain Catastrophizing Scale; ^2^ BPI: Brief Pain Inventory; ^3^ HSCL-20: Hopkins Symptom Checklist; depression severity, 20 items; ^4^ DUSOI: Duke Severity of Illness Inventory, physical comorbidity. *p <0.05; **p<0.01; ***p<0.001 | | | | | | | | | | | | |

**Table A2. Table of correlation coefficients (Spearman’s coefficient) between the constructs of interest at baseline.**
