# Supplementary material for: Short and long-term predictors of pain severity and interference in primary care patients with chronic musculoskeletal pain and depression
Source: BMC Musculoskelet Disord. 2023 Apr 5;24:270. doi: 10.1186/s12891-023-06357-2 (PMC10074832; doi:10.1186/s12891-023-06357-2)
Supplement: Supplementary file 2 — Supplementary Material 2 [file 12891_2023_6357_MOESM2_ESM.pdf]

The DROP Research Group.

Primary Care Area Camp de Tarragona, Catalan Health Institute.

Core research team

Enric Aragonès, Josep Lluís Piñol (†), Concepció Rambla, Catarina Tomé, Elisabet Sánchez, Antonia Caballero, Germán López-Cortacans, Jordi Miró.

Associate clinical group

Joan Josep Cabré (Sant Pere-1 Primary Care Centre, Reus), Joan Boj, Gemma Castellví, Maria Teresa Mauri (Sant Pere-2 Primary Care Centre, Reus), Meritxell Ricart, Joan Roca, Pere Claver Luque and Montserrat Romeu (Llibertat Primary Care Centre, Reus), Carmina Poblet, Jordi Jové, Antonia Caballero, Teresa Basora, Josep Vilalta and Carme Lucena (Horts de Miró Primary Care Centre, Reus), Mercedes Castro, Rosario Pérez, Yolanda Ortega, Marta Hernández, Irene Pascual, Eva Oya, Laia Guardia, Elisabet Salsench, Cinta De Diego, Cristina Ferrández and Mònica Maxenchs (Salou Primary Care Centre), Roxana Catalín, Arantxa Jara, Manuel Pérez, Fernando Gómez, Josep Maria Albiol, Belén Fernández and Núria Sarrà (Bonavista Primary Care Centre, Tarragona), Eva Satué, and Núria Ferrando (La Granja-Torreforta Primary Care Centre, Tarragona), Francesc Bobé, Sílvia Hernández, Olga Calviño, Laura Palacios, Laura Bascuas, Yvonne Fernández, Anna Moragas, Sílvia Crispi (Jaume I Primary Care Centre, Tarragona).
